# Supplementary material for: Screening of Entomopathogenic Fungal Culture Extracts with Honeybee Nosemosis Inhibitory Activity
Source: Insects. 2023 Jun 9;14(6):538. doi: 10.3390/insects14060538 (PMC10298890; doi:10.3390/insects14060538)
Supplement: Supplementary file 1 [file insects-14-00538-s001.zip › insects-2389506-supplementary.pdf]

Table S1. Entomopathogenic fungal isolates used in the experiment.

No. of isolate	Identification	No. of isolate	Identification	No. of isolate	Identification	No. of isolate	Identification
**1**	*Simplicillium aogashimaense*	**112**	*Metarhizium anisopliae*	**223**	*Cordyceps farinosa*	**327**	*Beauveria bassiana*
**2**	*Beauveria bassiana*	**113**	*Beauveria bassiana*	**224**	*Cordyceps fumosorosea*	**328**	*Beauveria bassiana*
**3**	*Beauveria brongniartii*	**114**	*Beauveria bassiana*	**225**	*Metarhizium lepidiotae*	**329**	*Metarhizium anisopliae*
**4**	*Tolypocladium album*	**115**	*Cordyceps farinosa*	**226**	*Pochonia bulbillosa*	**330**	*Metarhizium anisopliae*
**5**	*Beauveria bassiana*	**117**	*Pochonia bulbillosa*	**227**	*Paraconiothyrium sporulosum*	**331**	*Beauveria bassiana*
**6**	*Bionectria ochroleuca*	**119**	*Tolypocladium album*	**228**	*Metarhizium pemphigus*	**332**	*Beauveria bassiana*
**7**	*Beauveria bassiana*	**120**	*Metarhizium anisopliae*	**229**	*Beauveria bassiana*	**333**	*Metarhizium pemphigus*
**8**	*Tolypocladium album*	**121**	*Cordyceps farinosa*	**230**	*Beauveria bassiana*	**334**	*Beauveria bassiana*
**9**	*Beauveria bassiana*	**123**	*Tolypocladium album*	**231**	*Metarhizium anisopliae*	**335**	*Beauveria bassiana*
**10**	*Beauveria bassiana*	**124**	*Metarhizium anisopliae*	**232**	*Cordyceps javanica*	**336**	*Beauveria bassiana*
**12**	*Beauveria brongniartii*	**125**	*Metarhizium anisopliae*	**233**	*Cordyceps javanica*	**337**	*Beauveria bassiana*
**13**	*Beauveria brongniartii*	**126**	*Metarhizium anisopliae*	**234**	*Beauveria cf. bassiana*	**338**	*Beauveria bassiana*
**14**	*Pochonia rubescens*	**127**	*Paecilomyces lilacinus*	**235**	*Cordyceps javanica*	**339**	*Aspergillus versicolor*
**15**	*Tolypocladium cylindrosporum*	**128**	*Metarhizium anisopliae*	**236**	*Bionectria ochroleuca*	**340**	*Beauveria bassiana*
**16**	*Beauveria bassiana*	**129**	*Metarhizium anisopliae*	**237**	*Tolypocladium cylindrosporum*	**341**	*Beauveria bassiana*
**17**	*Beauveria bassiana*	**130**	*Beauveria bassiana*	**238**	*Metarhizium pemphigus*	**342**	*Metarhizium anisopliae*
**18**	*Cordyceps javanica*	**131**	*Paecilomyces lilacinus*	**239**	*Beauveria bassiana*	**343**	*Cordyceps javanica*
**19**	*Beauveria bassiana*	**132**	*Cordyceps javanica*	**240**	*Beauveria bassiana*	**344**	*Paecilomyces lilacinus*
**20**	*Beauveria bassiana*	**133**	*Beauveria bassiana*	**241**	*Bionectria ochroleuca*	**345**	*Aspergillus versicolor*
**22**	*Tolypocladium album*	**134**	*Metarhizium anisopliae*	**242**	*Lecanicillium sp.*	**346**	*Beauveria bassiana*
**23**	*Beauveria bassiana*	**135**	*Beauveria bassiana*	**243**	*Beauveria bassiana*	**347**	*Paecilomyces lilacinus*
**24**	*Paraconiothyrium sporulosum*	**136**	*Cordyceps javanica*	**244**	*Pochonia bulbillosa*	**348**	*Beauveria bassiana*
**25**	*Beauveria bassiana*	**137**	*Metarhizium anisopliae*	**245**	*Tolypocladium album*	**349**	*Metarhizium anisopliae*
**26**	*Beauveria bassiana*	**138**	*Metarhizium pemphigus*	**246**	*Metarhizium anisopliae*	**350**	*Bionectria ochroleuca*
**27**	*Beauveria bassiana*	**139**	*Metarhizium anisopliae*	**247**	*Beauveria bassiana*	**351**	*Beauveria bassiana*
**28**	*Paecilomyces lilacinus*	**140**	*Beauveria brongniartii*	**249**	*Beauveria bassiana*	**352**	*Clonostachys rosea*
**29**	*Beauveria bassiana*	**142**	*Myrothecium sp.*	**250**	*Tolypocladium cylindrosporum*	**353**	*Beauveria bassiana*
**30**	*Cordyceps javanica*	**143**	*Aspergillus lentulus*	**251**	*Metarhizium anisopliae*	**354**	*Beauveria bassiana*
**31**	*Fusarium oxysporum*	**144**	*Metarhizium anisopliae*	**252**	*Beauveria bassiana*	**355**	*Mucoromycotina sp.*
**32**	*Pochonia bulbillosa*	**145**	*Beauveria bassiana*	**253**	*Beauveria bassiana*	**356**	*Paecilomyces lilacinus*
**33**	*Pochonia bulbillosa*	**146**	*Beauveria bassiana*	**254**	*Beauveria brongniartii*	**357**	*Pochonia bulbillosa*
**34**	*Pochonia bulbillosa*	**148**	*Cordyceps farinosa*	**255**	*Beauveria bassiana*	**358**	*Beauveria bassiana*

**35**	*Beauveria bassiana*	**149**	*Beauveria bassiana*	**257**	*Metarhizium anisopliae*	**359**	*Beauveria brongniartii*
**37**	*Pochonia bulbillosa*	**150**	*Metarhizium anisopliae*	**258**	*Cordyceps farinosa*	**360**	*Metarhizium anisopliae*
**38**	*Paecilomyces marquandii*	**151**	*Metarhizium anisopliae*	**259**	*Metarhizium pemphigus*	**361**	*Cordyceps fumosorosea*
**39**	*Beauveria bassiana*	**152**	*Aspergillus lentulus*	**261**	*Beauveria bassiana*	**362**	*Aspergillus versicolor*
**40**	*Cordyceps fumosorosea*	**153**	*Metarhizium anisopliae*	**262**	*Acremonium strictum*	**363**	*Metarhizium anisopliae*
**41**	*Beauveria bassiana*	**154**	*Phialocephala sp.*	**263**	*Cordyceps farinosa*	**364**	*Paecilomyces marquandii*
**42**	*Beauveria bassiana*	**155**	*Metarhizium pemphigus*	**264**	*Beauveria bassiana*	**365**	*Metarhizium anisopliae*
**43**	*Cordyceps javanica*	**156**	*Metarhizium pemphigus*	**265**	*Beauveria bassiana*	**366**	*Beauveria bassiana*
**45**	*Bionectria ochroleuca*	**157**	*Metarhizium pemphigus*	**266**	*Metarhizium anisopliae*	**367**	*Metarhizium anisopliae*
**46**	*Aspergillus lentulus*	**158**	*Tolypocladium album*	**267**	*Verticillium insectorum*	**368**	*Metarhizium anisopliae*
**47**	*Cordyceps javanica*	**159**	*Metarhizium anisopliae*	**268**	*Metarhizium anisopliae*		
**48**	*Beauveria bassiana*	**160**	*Metarhizium anisopliae*	**269**	*Metarhizium pemphigus*		
**49**	*Beauveria bassiana*	**161**	*Beauveria bassiana*	**270**	*Beauveria bassiana*		
**50**	*Tolypocladium album*	**162**	*Cordyceps javanica*	**271**	*Metarhizium anisopliae*		
**51**	*Beauveria bassiana*	**163**	*Beauveria cf. bassiana*	**272**	*Cordyceps farinosa*		
**52**	*Beauveria bassiana*	**164**	*Beauveria bassiana*	**273**	*Simplicillium sp.*		
**53**	*Tolypocladium album*	**165**	*Cordyceps javanica*	**274**	*Metarhizium anisopliae*		
**54**	*Tolypocladium album*	**167**	*Lecanicillium sp.*	**275**	*Beauveria bassiana*		
**56**	*Cordyceps javanica*	**168**	*Beauveria bassiana*	**276**	*Metarhizium anisopliae*		
**57**	*Cordyceps javanica*	**169**	*Beauveria bassiana*	**277**	*Tolypocladium album*		
**58**	*Beauveria bassiana*	**170**	*Tolypocladium album*	**278**	*Metarhizium anisopliae*		
**59**	*Beauveria bassiana*	**171**	*Paecilomyces marquandii*	**279**	*Metarhizium anisopliae*		
**60**	*Pochonia bulbillosa*	**172**	*Myrothecium sp.*	**280**	*Pochonia bulbillosa*		
**61**	*Beauveria bassiana*	**173**	*Pochonia bulbillosa*	**281**	*Metarhizium anisopliae*		
**62**	*Beauveria bassiana*	**174**	*Beauveria bassiana*	**282**	*Beauveria bassiana*		
**63**	*Pochonia bulbillosa*	**175**	*Metarhizium anisopliae*	**283**	*Tolypocladium album*		
**64**	*Beauveria bassiana*	**177**	*Metarhizium anisopliae*	**284**	*Metarhizium anisopliae*		
**65**	*Beauveria bassiana*	**179**	*Metarhizium anisopliae*	**285**	*Cordyceps javanica*		
**66**	*Beauveria bassiana*	**180**	*Metarhizium pemphigus*	**286**	*Lecanicillium sp.*		
**67**	*Tolypocladium album*	**181**	*Cordyceps farinosa*	**287**	*Metarhizium anisopliae*		
**68**	*Tolypocladium album*	**182**	*Metarhizium anisopliae*	**288**	*Beauveria pseudobassiana*		
**69**	*Beauveria bassiana*	**183**	*Beauveria brongniartii*	**289**	*Beauveria bassiana*		
**70**	*Tolypocladium album*	**184**	*Metarhizium pemphigus*	**290**	*Metarhizium anisopliae*		
**71**	*Tolypocladium album*	**185**	*Pochonia bulbillosa*	**291**	*Tolypocladium album*		
**72**	*Beauveria bassiana*	**186**	*Metarhizium pemphigus*	**292**	*Beauveria pseudobassiana*		

**73**	*Tolypocladium album*	**187**	*Metarhizium pemphigus*	**293**	*Beauveria bassiana*		
**74**	*Beauveria bassiana*	**188**	*Beauveria bassiana*	**294**	*Beauveria bassiana*		
**75**	*Fusarium oxysporum*	**189**	*Beauveria bassiana*	**295**	*Metarhizium anisopliae*		
**76**	*Cordyceps javanica*	**190**	*Lecanicillium sp.*	**296**	*Metarhizium anisopliae*		
**77**	*Beauveria bassiana*	**192**	*Metarhizium pemphigus*	**297**	*Metarhizium anisopliae*		
**78**	*Pochonia bulbillosa*	**193**	*Beauveria bassiana*	**298**	*Cordyceps fumosorosea*		
**79**	*Beauveria bassiana*	**194**	*Metarhizium anisopliae*	**299**	*Metarhizium anisopliae*		
**80**	*Beauveria bassiana*	**195**	*Cordyceps fumosorosea*	**300**	*Beauveria pseudobassiana*		
**83**	*Paecilomyces lilacinus*	**196**	*Paecilomyces marquandii*	**301**	*Pochonia bulbillosa*		
**84**	*Myrothecium sp.*	**197**	*Metarhizium anisopliae*	**302**	*Metarhizium anisopliae*		
**85**	*Tolypocladium album*	**198**	*Metarhizium anisopliae*	**303**	*Beauveria bassiana*		
**86**	*Paecilomyces marquandii*	**199**	*Beauveria cf. bassiana*	**304**	*Myrothecium sp.*		
**87**	*Aspergillus lentulus*	**201**	*Beauveria bassiana*	**305**	*Beauveria bassiana*		
**88**	*Cordyceps javanica*	**202**	*Metarhizium anisopliae*	**306**	*Beauveria bassiana*		
**89**	*Beauveria bassiana*	**203**	*Metarhizium anisopliae*	**307**	*Beauveria bassiana*		
**90**	*Cordyceps javanica*	**204**	*Beauveria bassiana*	**308**	*Beauveria bassiana*		
**91**	*Beauveria bassiana*	**205**	*Bionectria ochroleuca*	**309**	*Beauveria bassiana*		
**92**	*Myrothecium sp.*	**206**	*Beauveria bassiana*	**310**	*Lecanicillium sp.*		
**93**	*Tolypocladium album*	**207**	*Metarhizium anisopliae*	**311**	*Metarhizium anisopliae*		
**95**	*Beauveria bassiana*	**208**	*Beauveria bassiana*	**312**	*Bionectria ochroleuca*		
**96**	*Beauveria bassiana*	**209**	*Metarhizium anisopliae*	**313**	*Lecanicillium sp.*		
**97**	*Cordyceps javanica*	**210**	*Beauveria bassiana*	**314**	*Beauveria pseudobassiana*		
**98**	*Simplicillium sp.*	**211**	*Metarhizium anisopliae*	**315**	*Cordyceps farinosa*		
**99**	*Beauveria bassiana*	**213**	*Beauveria brongniartii*	**316**	*Beauveria pseudobassiana*		
**100**	*Cordyceps javanica*	**214**	*Cordyceps fumosorosea*	**317**	*Cordyceps farinosa*		
**101**	*Pochonia bulbillosa*	**215**	*Beauveria bassiana*	**318**	*Metarhizium anisopliae*		
**102**	*Beauveria bassiana*	**216**	*Metarhizium anisopliae*	**320**	*Metarhizium anisopliae*		
**103**	*Metarhizium pemphigus*	**217**	*Metarhizium anisopliae*	**321**	*Beauveria bassiana*		
**104**	*Lecanicillium sp.*	**218**	*Metarhizium anisopliae*	**322**	*Beauveria bassiana*		
**105**	*Aspergillus lentulus*	**219**	*Beauveria bassiana*	**323**	*Metarhizium pemphigus*		
**106**	*Cordyceps javanica*	**220**	*Cordyceps farinosa*	**324**	*Tolypocladium album*		
**107**	*Tolypocladium album*	**221**	*Lecanicillium sp.*	**325**	*Beauveria bassiana*		
**110**	*Cordyceps javanica*	**222**	*Metarhizium anisopliae*	**326**	*Cordyceps farinosa*		

Table S2. Germination inhibition activity of entomopathogenic fungal culture extracts against *Nosema ceranae* spores. All fungal isolates used in the experiment were mostly only expressed with numbers for convenience

No. of isolate	Inhibition rate (%)	Standard error	No. of isolate	Inhibition rate (%)	Standard error	No. of isolate	Inhibition rate (%)	Standard error	No. of isolate	Inhibition rate (%)	Standard error
**Control**	0.00	1.24	**290**	77.84	4.85	**104**	64.47	13.91	**194**	21.49	2.95
**296**	96.45	2.81	**25**	77.73	3.03	**146**	64.16	4.47	**186**	21.05	5.59
**329**	96.10	3.73	**357**	77.68	2.92	**217**	63.41	5.43	**190**	20.07	2.74
**293**	94.42	2.36	**361**	77.56	3.93	**42**	63.20	1.11	**160**	17.72	3.77
**242**	94.00	3.39	**272**	77.44	2.79	**269**	62.36	4.55	**78**	17.67	3.94
**189**	93.66	5.88	**16**	77.26	3.39	**171**	62.18	2.74	**63**	17.11	2.55
**320**	93.66	2.92	**246**	76.83	5.40	**270**	62.05	8.07	**86**	17.11	2.74
**181**	93.61	2.27	**258**	76.83	2.44	**62**	61.62	3.20	**83**	16.82	1.19
**298**	93.41	2.01	**263**	76.83	1.94	**20**	61.51	1.71	**158**	16.67	5.15
**306**	93.19	5.84	**303**	76.77	3.82	**224**	60.98	1.54	**214**	16.10	1.58
**300**	93.08	2.59	**338**	76.60	3.99	**110**	60.69	3.86	**3**	15.53	4.40
**115**	92.47	3.93	**363**	76.59	3.77	**85**	60.53	2.88	**75**	15.53	2.50
**317**	91.22	2.12	**247**	76.51	2.00	**312**	60.15	2.35	**192**	15.41	3.93
**347**	90.98	5.48	**105**	76.38	0.98	**173**	59.87	3.14	**33**	15.38	3.33
**135**	90.94	1.95	**8**	76.29	3.82	**336**	59.67	5.20	**46**	13.16	2.27
**79**	90.76	5.48	**349**	76.26	2.44	**103**	58.60	4.62	**205**	12.37	3.93
**206**	90.60	6.78	**210**	75.88	3.33	**234**	58.60	2.61	**84**	11.89	3.33
**144**	90.48	2.18	**52**	75.83	1.69	**241**	58.54	5.20	**56**	11.48	7.63
**341**	90.43	3.61	**65**	75.76	2.79	**308**	57.51	3.32	**139**	11.18	1.57
**67**	90.15	2.36	**209**	75.74	2.44	**9**	56.75	2.76	**93**	9.77	2.59
**188**	90.15	1.11	**275**	75.73	1.94	**199**	56.62	4.28	**31**	9.21	2.00
**243**	89.91	7.87	**222**	75.61	3.60	**257**	56.59	4.92	**53**	9.18	8.97
**208**	89.67	13.18	**254**	75.61	7.08	**203**	56.58	4.47	**32**	7.63	3.93
**265**	89.64	8.29	**342**	75.54	8.36	**351**	56.30	2.21	**43**	7.60	3.61
**117**	89.57	6.10	**19**	75.47	0.63	**266**	56.10	6.69	**128**	6.64	3.93
**220**	89.49	1.58	**76**	75.46	4.99	**297**	56.10	3.18	**187**	1.67	2.86
**253**	89.40	2.93	**4**	75.21	9.32	**226**	55.12	4.94	**45**	0.00	3.50
**60**	89.29	3.72	**331**	75.16	5.17	**267**	55.12	3.43	**14**	0.00	2.88
**309**	89.27	2.34	**288**	75.12	3.74	**126**	54.87	6.07	**6**	0.00	3.33
**294**	89.10	2.68	**311**	75.12	3.14	**182**	54.74	2.93	**18**	0.00	3.20
**287**	89.02	4.09	**245**	75.06	6.18	**148**	54.64	4.03	**22**	0.00	5.66

**161**	88.66	2.86	**40**	75.01	2.18	**323**	54.63	4.12	**259**	0.00	6.35
**289**	88.44	3.65	**195**	74.91	3.50	**134**	54.34	7.09	**154**	0.00	1.42
**95**	88.39	2.56	**360**	74.63	5.46	**70**	53.76	3.45	**37**	0.00	1.51
**276**	88.29	4.78	**2**	74.31	8.52	**316**	53.66	7.39	**34**	0.00	5.15
**249**	88.20	10.95	**41**	74.08	4.87	**324**	53.31	7.50	**71**	0.00	8.92
**64**	88.10	1.66	**57**	74.07	3.83	**168**	53.15	5.07	**28**	0.00	3.39
**354**	87.36	4.72	**344**	74.04	3.89	**221**	52.44	2.79	**54**	0.00	4.40
**340**	87.15	2.73	**211**	73.80	2.65	**228**	52.20	4.77	**138**	0.00	4.43
**348**	86.64	3.67	**172**	73.78	4.62	**299**	52.20	6.64	**13**	0.00	4.91
**26**	86.62	2.86	**327**	73.69	7.67	**278**	51.30	3.51			
**165**	86.43	1.24	**271**	73.66	4.06	**136**	51.15	2.37			
**96**	86.37	10.47	**280**	73.66	2.68	**202**	51.08	5.30			
**367**	86.30	3.11	**193**	73.57	2.39	**23**	50.65	5.59			
**121**	85.91	5.93	**29**	73.56	6.07	**49**	50.00	4.50			
**17**	85.85	2.51	**30**	73.44	3.42	**157**	49.82	5.63			
**204**	85.84	1.75	**215**	73.42	2.34	**98**	49.74	1.85			
**59**	85.83	3.14	**125**	73.23	1.75	**237**	48.58	2.92			
**97**	85.39	3.50	**365**	72.93	1.85	**91**	46.68	3.73			
**235**	85.39	4.17	**7**	72.71	1.55	**80**	46.29	2.90			
**330**	85.37	2.43	**286**	72.56	6.98	**102**	46.28	4.15			
**333**	85.37	3.02	**302**	72.56	3.93	**155**	45.85	4.12			
**339**	85.37	4.55	**368**	72.52	3.93	**310**	45.84	2.72			
**51**	85.21	3.27	**39**	72.50	3.33	**283**	45.64	4.28			
**164**	85.19	4.75	**5**	72.40	5.86	**120**	45.49	2.05			
**1**	84.88	0.31	**201**	72.22	4.40	**264**	45.42	6.06			
**183**	84.67	4.91	**177**	72.01	6.62	**282**	45.12	5.37			
**145**	84.63	7.80	**143**	71.54	4.75	**366**	44.80	2.64			
**27**	84.26	1.16	**163**	71.32	11.06	**151**	44.74	3.72			
**321**	84.21	3.82	**77**	71.25	2.56	**315**	44.39	2.14			
**364**	84.15	5.17	**47**	71.03	3.93	**129**	44.08	4.12			
**261**	84.13	2.61	**174**	70.92	6.07	**127**	44.05	3.30			
**218**	83.90	4.12	**350**	70.89	1.17	**101**	43.95	3.93			
**279**	83.90	4.08	**233**	70.89	3.51	**175**	43.82	6.19			
**307**	83.87	1.73	**346**	70.77	3.32	**213**	43.42	3.14			
**90**	83.85	3.93	**281**	70.73	4.36	**335**	42.92	3.43			
**137**	83.48	3.83	**326**	70.73	8.77	**232**	41.46	6.06			

**58**	83.40	4.33	**69**	70.49	5.76	**313**	41.45	4.41			
**61**	82.98	2.96	**334**	70.49	5.95	**106**	40.82	5.69			
**358**	82.95	6.21	**132**	70.22	8.22	**304**	40.79	4.30			
**227**	82.86	3.44	**114**	70.20	3.11	**359**	40.49	1.92			
**328**	82.68	3.00	**229**	69.80	3.02	**89**	40.47	2.27			
**99**	82.64	3.20	**24**	69.75	5.98	**162**	40.26	4.99			
**72**	82.64	5.76	**322**	69.69	2.62	**292**	39.65	5.39			
**184**	82.39	6.00	**352**	69.27	3.57	**179**	39.21	3.93			
**10**	82.26	1.08	**268**	69.07	4.55	**325**	38.70	3.22			
**274**	82.23	1.92	**197**	69.05	5.11	**362**	38.43	3.77			
**150**	82.14	4.46	**152**	69.05	3.39	**225**	36.36	8.61			
**239**	81.94	2.86	**170**	69.04	2.77	**207**	35.66	3.35			
**35**	81.83	5.59	**68**	68.67	7.31	**131**	35.49	3.83			
**337**	81.61	8.08	**74**	68.53	4.40	**180**	35.26	3.11			
**238**	80.98	3.11	**262**	68.29	4.15	**305**	34.34	1.97			
**284**	80.98	2.55	**250**	68.29	3.25	**140**	34.21	1.95			
**295**	80.76	4.12	**153**	68.19	2.68	**50**	33.88	3.33			
**332**	80.64	6.75	**113**	67.59	1.78	**159**	33.88	3.93			
**185**	80.58	1.25	**353**	67.07	4.66	**156**	33.33	7.80			
**255**	80.49	4.72	**198**	66.75	4.91	**88**	32.65	6.00			
**252**	80.42	9.18	**133**	66.62	2.51	**231**	32.21	3.11			
**230**	80.15	2.86	**285**	66.54	5.17	**119**	32.02	4.15			
**100**	80.09	6.44	**244**	66.50	5.24	**87**	30.64	3.83			
**240**	79.97	3.59	**48**	66.33	7.12	**223**	29.52	5.20			
**318**	79.74	5.46	**355**	66.18	2.62	**38**	28.95	3.33			
**291**	79.30	3.87	**251**	65.75	2.28	**66**	27.33	4.15			
**149**	79.11	8.14	**169**	65.72	5.17	**277**	26.83	3.57			
**107**	78.67	5.48	**314**	65.61	4.71	**236**	25.37	2.93			
**130**	78.60	5.25	**142**	65.58	3.93	**73**	25.36	1.08			
**219**	78.47	2.59	**12**	65.26	4.99	**196**	24.89	3.33			
**124**	78.46	3.33	**92**	64.98	3.20	**167**	24.21	2.27			
**216**	78.16	1.16	**273**	64.88	2.94	**112**	23.68	5.55			
**301**	78.05	2.20	**15**	64.73	2.88	**345**	23.63	3.37			
**343**	78.05	7.04	**356**	64.63	2.62	**123**	23.03	6.00			

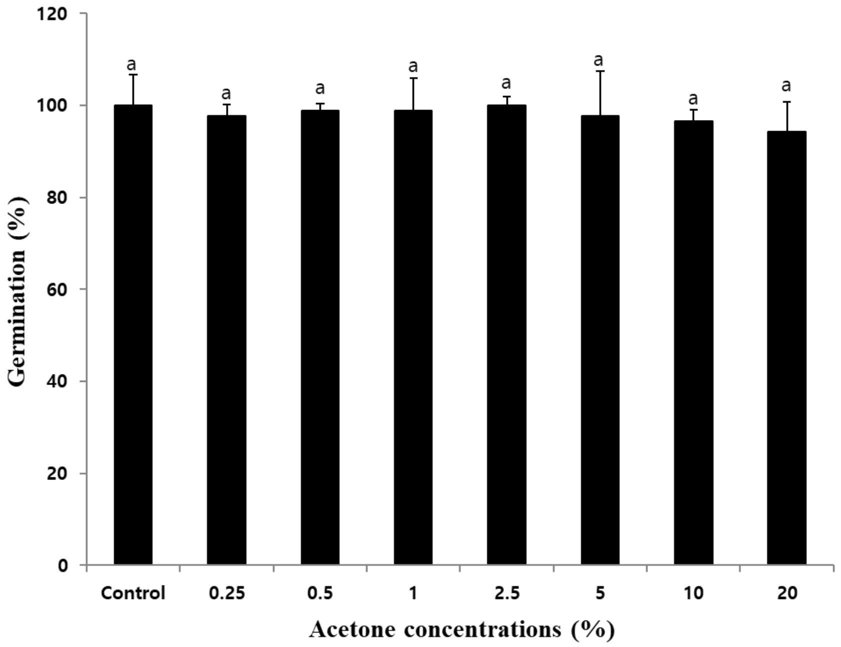

Figure S1. Viability of *Nosema ceranae* spores by acetone treatment at different concentrations. After treating the spores with acetone at each concentration, the germination rate was determined by an *in vitro* germination assay. Values with different letters are significantly different ($p < 0.05$, SNK test in one-way ANOVA).

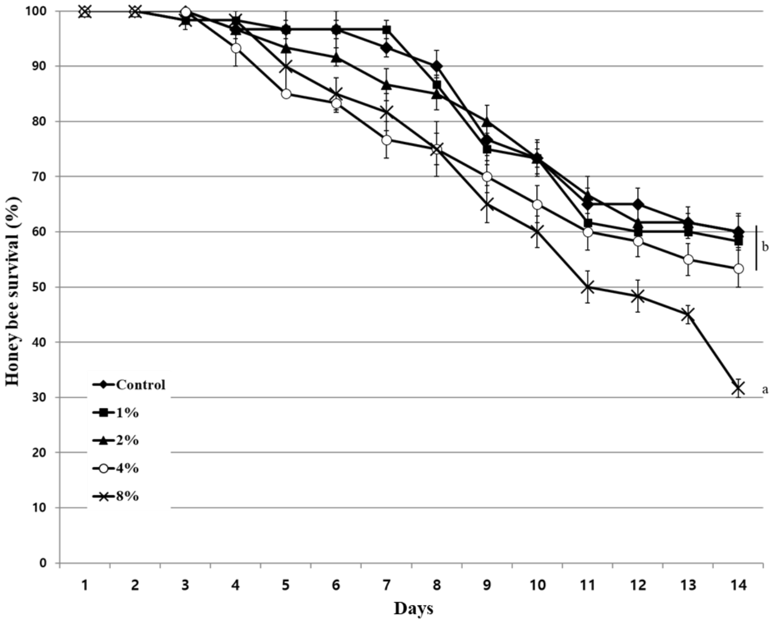

Figure S2. Honey bee survival by acetone treatment at different concentrations. A mixture of acetone and 50% sucrose solution was fed to honey bees. After that, the survival rate of honey bees was determined for 14 days. The control group was fed only a 50% sucrose solution. Data show the mean ± SE. Values with different letters are significantly different ($p < 0.05$, SNK test in one-way ANOVA) at 14 days.
